# Five-Year Efficacy and Safety of the FRED Flow-Diverting Stent for Intracranial Aneurysms: Results From a Prospective Cohort

**DOI:** 10.1161/SVIN.125.002187

**Published:** 2026-04-02

**Authors:** Leopoldo Guimaraens, Elio Vivas, Jesus Saldaña, Rafael Mercedes-Terrero, Ernest Balaguer, Dunia Mon, Elisa Cuadrado-Godia, Joan Jiménez-Balado, Angel Ois, Daniel Guisado-Alonso

**Affiliations:** 1 J.J. Merland Department of Therapeutic Neuroangiography, Hospital Universitari General de Catalunya and Hospital del Mar, Barcelona, Spain (L.G., E.V., J.S., R.M.-T.).; 2 Department of Neurology, Hospital del Mar and Universitat Pompeu Fabra, Barcelona, Spain (E.C.-G., A.O., D.G.-A.).; 3 Department of Neurology, Hospital Universitari General de Catalunya, Barcelona, Spain (E.B., D.M.).; 4 Neurovascular Research Group, Hospital del Mar Medical Research Institute, Barcelona, Spain (E.C.-G., J.J.-B., A.O., D.G.-A.).

**Keywords:** angiography, digital subtraction, cohort studies, follow-up studies, intracranial aneurysm, stents, treatment outcome

## Abstract

**BACKGROUND:**

Flow diversion is a well-established endovascular treatment for intracranial aneurysms, yet long-term outcome data remain scarce. Our aim was to report efficacy and safety at 5 years for the flow redirection endoluminal device in a consecutive cohort with protocolized follow-up.

**METHODS:**

All consecutive patients with intracranial aneurysms treated with a flow redirection endoluminal device between December 2015 and July 2020 at 2 university hospitals were included in a prospective registry. Treatment decisions and procedural protocols were standardized. Clinical and imaging follow-up, including scheduled 3-dimensional rotational digital subtraction angiography, continued through August 2025. The primary end point was complete or near-complete occlusion at 5 years, defined as O’Kelly-Marotta (angiographic grading scale) grades C to D. Ischemic, hemorrhagic, and device-related events were prospectively recorded.

**RESULTS:**

A total of 203 patients (mean age, 54.7 years; 77.3% women) with 236 intracranial aneurysms were analyzed. At 5 years, complete or near-complete occlusion (O’Kelly-Marotta grades C–D) was achieved in 225 aneurysms (95.3%), comprising 203 of 211 (96.2%) in the anterior circulation and 22 of 25 (88.0%) in the posterior circulation. Retreatment was required in 5 aneurysms (2.1%), all treated before 2019. Symptomatic complications occurred in 12 of 203 patients (5.9%): 10 ischemic (4.9%) and 2 hemorrhagic (1.0%); no treatment-related deaths, rebleeding, or delayed rupture occurred. One patient had moderate permanent disability, and one died from a contralateral hypertensive hemorrhage deemed unrelated to the device. Asymptomatic device-related findings occurred in 22 of 236 aneurysms (9.3%).

**CONCLUSIONS:**

Flow redirection endoluminal device achieved durable aneurysm occlusion with a low rate of clinically relevant complications. These findings support sustained efficacy and safety across diverse aneurysm morphologies and locations.

## GRAPHIC ABSTRACT

**Figure:**
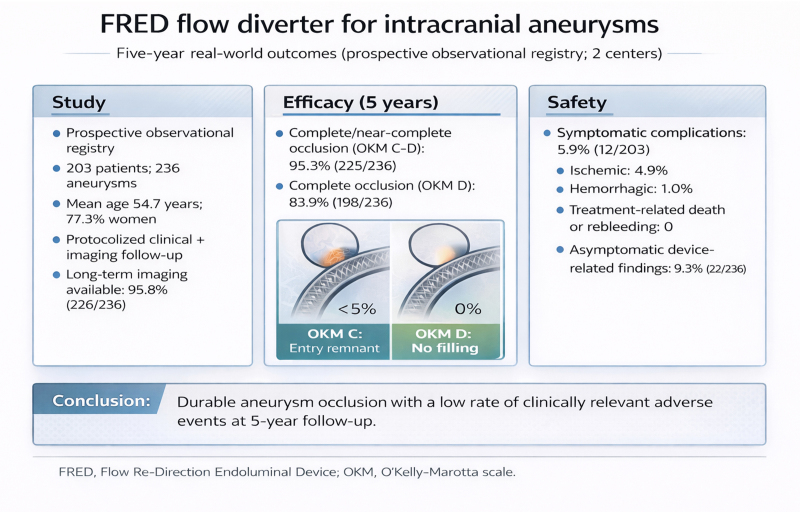

**CLINICAL PERSPECTIVE**

**What Is New?**

- In a consecutive, prospectively followed cohort with protocolized imaging surveillance including 3-dimensional rotational angiography, the flow redirection endoluminal device achieved durable 5-year aneurysm occlusion with a low rate of clinically relevant complications.

**What Are the Clinical Implications?**

- These long-term real-world data support the sustained safety and efficacy of the flow redirection endoluminal device and may help inform follow-up strategies in routine practice.

Flow-diverting stents have transformed the treatment of intracranial aneurysms, offering an effective option for wide-necked, complex, or previously untreatable lesions.^1–3^ The dual-layer flow redirection endoluminal device (FRED; MicroVention) was designed to promote aneurysm occlusion while preserving parent vessel patency. Although short- and mid-term outcomes have been favorable,^4^ evidence on long-term outcomes remains limited.^5^ Our aim was to assess efficacy and safety at 5 years of FRED in a consecutive cohort with protocolized follow-up.

## Methods

Anonymized data that support the findings of this study are available from the corresponding author on reasonable request. This study is reported in accordance with the STROBE reporting guidelines (Strengthening the Reporting of Observational Studies in Epidemiology; Supplemental Material).

### Study Design and Population

We conducted a prospective observational registry including all consecutive patients with intracranial aneurysms treated with FRED (including FRED junior; the surface-modified FRED was not available during the inclusion period) between December 2015 and July 2020 at 2 university hospitals. All procedures were performed by the same neurointerventional team. Clinical and imaging follow-up was scheduled through August 2025 to ensure 5-year data per patient. Cases with incomplete follow-up (due to death, loss to follow-up, or retreatment of the target aneurysm) were prospectively identified and documented. For aneurysms without 5-year imaging due to death or loss to follow-up, long-term occlusion status was determined using the most recent available follow-up imaging before censoring. Efficacy and device-related findings were analyzed per aneurysm; clinical complications were analyzed per patient.

### Procedural Protocol

Treatment decisions were made by senior interventional neuroradiologists based on aneurysm morphology in cases where conventional endovascular options were unsuitable. Indications for FRED included wide-neck saccular aneurysms (fundus-to-neck ratio <2 or neck diameter >4 mm), fusiform, dissecting, blister-like, giant aneurysms (>25 mm), or recanalized lesions after previous coiling, surgery, or stenting. Patients were excluded if the parent vessel had an unfavorable branch configuration (eg, a branch arising at an acute angle from the segment to be covered) or if the parent artery diameter was <2 mm. All patients received dual antiplatelet therapy (aspirin 300 mg and clopidogrel 75 mg daily) for at least 5 days before the procedure, with platelet inhibition confirmed by VerifyNow assay, as in previous studies.^4,6,7^ Procedures were performed under general anesthesia with systemic anticoagulation (initial heparin 50 IU/kg, followed by 1000 IU boluses to maintain an activated clotting time of 250–300 seconds). Device delivery was performed via transfemoral coaxial access. Three-dimensional rotational digital subtraction angiography (3D-DSA) was obtained in all cases to assess parent artery anatomy and guide sizing. Stent diameter was selected to match the largest parent artery diameter, with 25% to 30% oversizing for wall apposition when required. Length was chosen to ensure full neck coverage with 2.5 to 3 mm margins proximally and distally, following published protocols.^4,6,7^ FRED junior was preferentially selected for smaller-caliber parent vessels (typically <3 mm) and more distal anatomies in accordance with manufacturer sizing recommendations. The stent was delivered through a microcatheter over a 0.016-inch microguidewire using a 6F guide catheter system. If positioning was suboptimal, the stent could be resheathed and repositioned, provided <80% had been deployed. Dual antiplatelet therapy was continued for at least 6 months, followed by single antiplatelet therapy for 18 to 24 months.

### Data Collection and Follow-Up

All patients underwent neurological evaluation by vascular neurologists at baseline and at discharge. Clinical follow-up was scheduled every 3 to 6 months for up to 5 years and coordinated jointly by the neurology and neurointerventional teams. Neurological events or complications during follow-up were adjudicated by neurologists independent of the index procedure. Vascular imaging followed predefined time points: catheter angiography with 3D-DSA immediately after the procedure, at 3 to 9 months, and annually thereafter, in line with published protocols.^2,6,7^ Where appropriate, noninvasive magnetic resonance (MR) angiography or computed tomography (CT) angiography supplemented catheter studies. A 5-year digital subtraction angiography was planned for all patients unless contraindicated or declined. All follow-up imaging studies were reviewed in scheduled multidisciplinary meetings. Final imaging outcomes were adjudicated by consensus between at least 2 senior interventional neuroradiologists.

### Outcome Measures

The primary efficacy outcome was aneurysm occlusion on follow-up imaging, graded with the O’Kelly-Marotta (angiographic grading scale; OKM) scale (A total filling; B subtotal filling; C entry remnant; D no filling). Successful treatment was defined as complete or near-complete occlusion (OKM grades C–D) at 5 years. OKM grades were prospectively recorded at each imaging time point, and final occlusion status was adjudicated at 5 years using digital subtraction angiography in most cases, with CT or MR angiography where clinically indicated.

Safety outcomes comprised all adverse events and asymptomatic device-related findings. Symptomatic complications were events resulting in new neurological symptoms or deficits attributed to ischemia or hemorrhage. Asymptomatic device-related findings on imaging included in-stent stenosis, parent artery occlusion, device malposition or deformation, and aneurysm growth. Functional outcomes were assessed with the modified Rankin Scale. Neurological events were confirmed by vascular neurologists independent of the index procedure, and imaging and technical findings were assessed by consensus between at least 2 senior interventional neuroradiologists from the treating team. Retreatment of the target aneurysm during follow-up was recorded. By prespecified criteria, aneurysms that underwent retreatment were classified as incomplete occlusion in the main analysis irrespective of the final angiographic result.

### Statistical Analysis

No formal sample size calculation was performed because the study size was determined by consecutive eligible patients treated during the prespecified inclusion period; analyses are descriptive and exploratory. Continuous variables are presented as mean (SD) or median (interquartile range), and categorical variables as counts and percentages. Analyses of clinical complications were performed per patient (N=203), whereas efficacy outcomes and device-related findings were analyzed per aneurysm (N=236). Group comparisons used the χ^2^ test or Fisher exact test for categorical variables and the *t* test or Mann-Whitney *U* test for continuous variables, as appropriate. Missing data were not imputed. Analyses were performed using available data, and denominators are reported where applicable. A complete-case sensitivity analysis excluding aneurysms without 5-year imaging was also performed. Analyses were performed with R (R Foundation for Statistical Computing). Statistical significance was set at *P*<0.05. Reporting follows STROBE guidelines.

### Ethics Statement

The study was approved by the Ethics Committee of Hospital del Mar, Barcelona, Spain (Comité de Ética de la Investigación con Medicamentos, CEIm-PSMAR; 2008/3083/l), and conducted in accordance with the Declaration of Helsinki. Written informed consent was obtained from all participants.

## Results

A total of 203 patients (mean age, 54.7 years, SD 11.6; range, 15–81) underwent FRED stent placement for 236 intracranial aneurysms. Women comprised 157 of 203 (77.3%) of the cohort. In total, 217 stents were implanted: 198 of 217 (91.2%) for a single aneurysm and 19 of 217 (8.8%) for 2 contiguous aneurysms.

Long-term follow-up was available for 226 of 236 aneurysms (95.8%). Follow-up was incomplete in 10 of 236 cases (4.2%): 5 underwent retreatment (median, 24 months [interquartile range, 20–26]), 3 patients died from causes unrelated to aneurysm or endovascular therapy, and 2 aneurysms in a single patient were lost to follow-up. In the 5 aneurysms without 5-year imaging due to death or loss to follow-up, the most recent available follow-up imaging (median, 35 months [interquartile range, 30–48]) showed adequate occlusion. All retreatments occurred in aneurysms treated between 2016 and 2018; none were performed thereafter. In a complete-case sensitivity analysis excluding aneurysms without 5-year imaging, adequate occlusion remained similar (220/231, 95.2%). Among the 226 aneurysms with complete follow-up, 5-year imaging was obtained in all cases, with digital subtraction angiography in most and CT or MR angiography in selected cases. In 19 cases, a 5-year 3D-DSA was not performed, most often due to patient preference or logistical reasons; 5-year imaging in these cases was by CT or MR angiography. Baseline characteristics are summarized in Table 1. The peak year of treatment was 2018 (67/236, 28.4%), without statistically significant differences across years (*P*=0.518). Most aneurysms were saccular (107/236, 45.3%), followed by recanalized (67/236, 28.4%), fusiform (23/236, 9.8%), blister (20/236, 8.5%), dissecting (8/236, 3.4%), and aneurysmal dilatations (11/236, 4.7%). Rupture at presentation was present in 109 of 236 (46.2%). The most frequent treatment indications were wide-necked morphology (107/236, 45.3%) and recanalization (67/236, 28.4%). Nearly half (116/236, 49.2%) were small aneurysms, whereas medium (32/236, 13.6%), large (12/236, 5.1%), and giant (2/236, 0.9%) were less common; size was not applicable in 74 of 236 (31.4%) due to complex morphology. Most aneurysms were in the anterior circulation (211/236, 89.4%), whereas 25 of 236 (10.6%) were located in the posterior circulation. FRED junior was used in 49 of 236 (20.8%) according to the sizing-based selection criteria described in the Methods. Immediate postprocedural OKM grading showed partial filling (A–B) in 199 of 236 (84.3%) and neck remnant or complete occlusion (C–D) in 37 of 236 (15.7%).

**Table 1.**
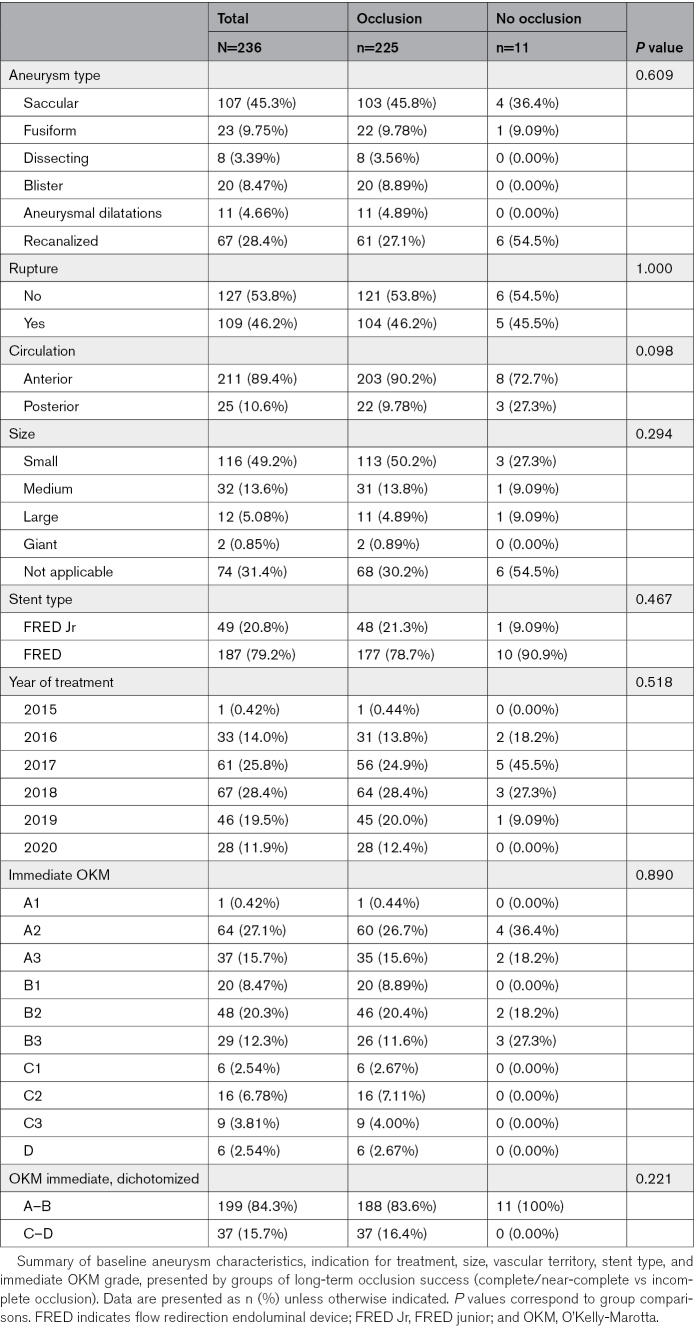
Baseline Characteristics of Aneurysms According to 5-Year Occlusion Status

### Efficacy Outcomes

At 5 years, complete or near-complete occlusion (OKM grades C–D) was achieved in 225 of 236 aneurysms (95.3%), whereas incomplete occlusion persisted in 11 of 236 (4.7%). In the anterior circulation, adequate occlusion occurred in 203 of 211 (96.2%) and complete occlusion in 175 of 211 (82.9%). In the posterior circulation, adequate occlusion was achieved in 22 of 25 (88.0%) and complete occlusion in 18 of 25 (72.0%). The final distribution of OKM grades was: D 193 (81.8%), C3 11 (4.7%), C2 9 (3.8%), C1 12 (5.1%), B3 6 (2.5%), B2 2 (0.8%), B1 1 (0.4%), A3 1 (0.4%), and A2 1 (0.4%; Table 1). Among aneurysms with incomplete occlusion, only 1 showed progression (C2–B3); the remainder remained stable. As shown in Table 1, no significant associations were observed between long-term occlusion status and year of treatment, aneurysm type, rupture, indication, size, stent type, or immediate OKM grade (all *P*>0.05). Posterior-circulation aneurysms showed a higher rate of nonocclusion at last follow-up (12.0% [3/25] versus 3.8% [8/211] in the anterior circulation), but this difference did not reach statistical significance (*P*=0.098). Immediate postprocedural OKM grade was not predictive of long-term occlusion (*P*=0.89). Among the 37 aneurysms with immediate near-complete or complete occlusion (OKM grades C–D, 15.7%), none worsened during follow-up. Five aneurysms underwent additional endovascular retreatment due to suboptimal occlusion, with a median time to retreatment of 24 months (interquartile range, 20–26). By prespecified criteria, these were classified as incomplete occlusion in the main analysis, although all achieved complete occlusion at 5 years. If classified according to their final angiographic outcome, the complete occlusion rate would rise to 198 of 236 (83.9%), and adequate occlusion (C–D) to 230 of 236 (97.5%).

### Safety Outcomes

Symptomatic complications occurred in 12 of 203 patients (5.9%): 10 of 203 ischemic (4.9%) and 2 of 203 hemorrhagic (1.0%; Table 2). Of these, 1 patient had moderate disability at follow-up (modified Rankin Scale score 3). One immediate hemorrhage caused no lasting deficit, and 1 delayed fatal contralateral hypertensive intracerebral hemorrhage under dual antiplatelet therapy was adjudicated as unrelated to the device. No treatment-related deaths, rebleeding, or delayed rupture occurred.

**Table 2.**
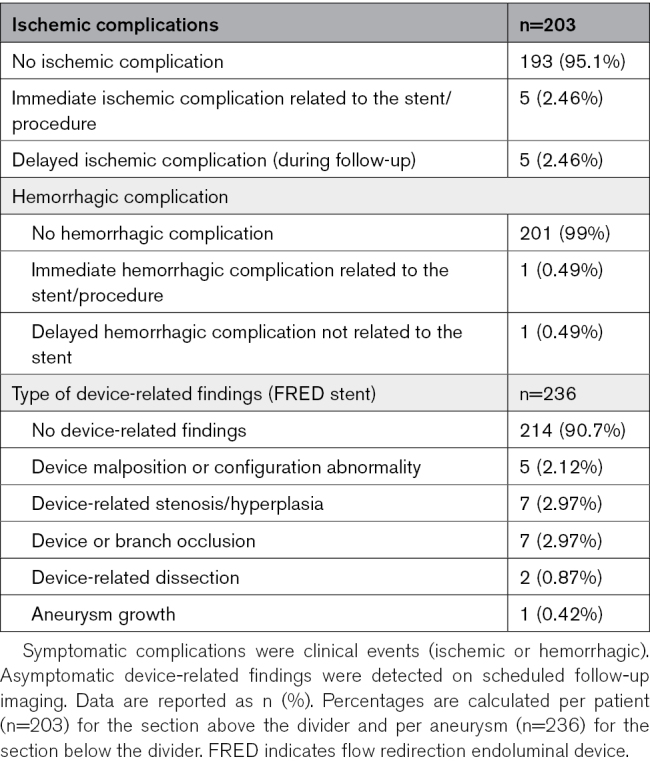
Summary of Adverse Events During Follow-Up

Asymptomatic device-related findings were observed in 22 of 236 aneurysms (9.3%). These findings were identified on scheduled follow-up imaging and were managed conservatively on a case-by-case basis; most were managed with clinical/imaging surveillance, with no additional procedures except for the single balloon angioplasty case already reported. These included device or branch occlusion (7/236, 3.0%), stenosis or hyperplasia (7/236, 3.0%), malposition or configuration abnormality (5/236, 2.1%), dissection (2/236, 0.8%), and aneurysm growth (1/236, 0.4%; Table 2). None caused clinical symptoms. One case of stenosis underwent balloon angioplasty despite being asymptomatic.

### Outcomes by Location

Territory-specific 5-year occlusion outcomes (anterior versus posterior circulation) are reported in the Efficacy Outcomes section; Table 3 details outcomes by individual aneurysm location. Among 236 aneurysms, the most frequent location was the carotid siphon (109/236, 46.2%), followed by the middle cerebral artery (32/236, 13.6%), posterior communicating artery (25/236, 10.6%), and anterior communicating artery (19/236, 8.1%; Table 3). Other sites included the anterior cerebral artery (10/236, 4.2%), basilar terminus (9/236, 3.8%), vertebral artery (7/236, 3.0%), anterior choroidal artery (6/236, 2.5%), posterior inferior cerebellar artery (5/236, 2.1%), cervical carotid (5/236, 2.1%), carotid terminus (5/236, 2.1%), and posterior cerebral artery (4/236, 1.7%). Adequate occlusion rates were consistently high across most locations: 106 of 109 (97.2%) in the carotid siphon, 30 of 32 (93.8%) in the middle cerebral artery, and 100% in several anterior circulation sites (Table 3). The lowest rate was in posterior inferior cerebellar artery aneurysms (3/5, 60.0%; Table 3). Symptomatic ischemic and hemorrhagic events were observed in the anterior circulation. Device-related findings were more frequent in the posterior fossa (40.0% in posterior inferior cerebellar artery, 22.2% in basilar terminus) compared with the anterior circulation (11.9% in the carotid siphon, 12.5% in the middle cerebral artery), although this difference was not statistically significant (*P*=0.27; Table 3). All device-related events were asymptomatic and detected only on imaging (Table 3).

**Table 3.**
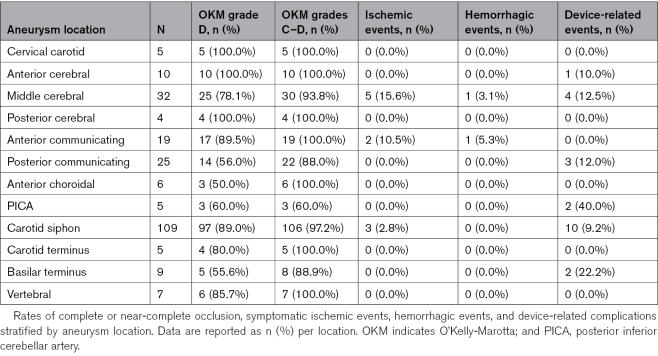
Outcomes by Aneurysm Location

## Discussion

This prospective series represents the largest consecutive cohort to date of patients treated with FRED for ruptured and unruptured intracranial aneurysms with protocolized 5-year follow-up, demonstrating high and durable rates of adequate aneurysm occlusion (95.3%; OKM grades C–D) and a favorable long-term safety profile across a broad range of aneurysm morphologies and vascular territories.

Numerous studies have reported long-term outcomes of flow diverters for the treatment of intracranial aneurysms^8–10^; however, long-term in most series refers to follow-up periods beyond 1 year, with mean follow-up usually limited to 2 or 3 years.^2,3,8,9^ Studies providing outcomes at ≥5 years remain scarce and typically include relatively limited case numbers. Among the available literature, the Pipeline Embolization Device is the most extensively studied flow diverter, as it was among the earliest introduced into clinical practice. Five-year or longer outcomes have been reported in a recent meta-analysis^9^ and several large registries/trials, including the single-center registry of 1000 aneurysms^11^ and Pipeline for Uncoilable or Failed Aneurysms study.^12^ Compared with these long-term Pipeline Embolization Device data, our results with FRED demonstrate comparable efficacy in both anterior and posterior circulations. In our cohort of all consecutive aneurysms with 5-year follow-up, complete occlusion (OKM grade D) was achieved in 175 of 211 (82.9%) in the anterior circulation, with adequate occlusion (OKM grades C–D) in 203 of 211 (96.2%). In the posterior circulation (n=25), complete occlusion was achieved in 18 of 25 (72.0%) and adequate occlusion in 22 of 25 (88.0%). Several methodological differences complicate direct comparisons across studies. Notably, in several long-term Pipeline Embolization Device series, only a fraction of eligible aneurysms underwent DSA-based assessment at 5 years, introducing potential selection bias, and retreatment handling in the final efficacy analysis is not always clearly specified.^11–13^ In our study, retreated aneurysms were conservatively classified as incomplete occlusion in the main analysis; if classified according to their final angiographic result at 5 years, the rate of adequate occlusion would increase to 97.5%. Furthermore, angiographic outcomes in key Pipeline Embolization Device studies were assessed using the Raymond–Roy classification, which differs from the OKM scale employed systematically in our study, and Pipeline for Uncoilable or Failed Aneurysms study was restricted to large, complex internal carotid artery aneurysms and used strict inclusion criteria and independent core laboratory adjudication.^12^

Long-term data specific to FRED remain limited. The largest published long-term series to date, reported by Hohenstatt et al,^5^ included 53 aneurysms (mean follow-up, 69.2 months) and found complete occlusion in 85% and adequate occlusion in 100%, without late recanalizations, retreatments, or treatment-related neurological complications or deaths; imaging follow-up relied mainly on MR imaging, reserving 3D-DSA for equivocal findings or suspected residual aneurysm filling. A multicenter study of 155 aneurysms treated with FRED, with follow-up up to 75 months (median, 36 months), did not provide outcomes at 5 years, but reported a final complete occlusion rate of 91.9% and a retreatment rate of 2%, with only 1 device-related complication observed after 24 months.^14^ Our findings extend these data by providing protocolized 5-year outcomes in a larger consecutive cohort with systematic OKM grading and predominantly 3D-DSA follow-up, offering a robust long-term benchmark for FRED performance. Our results also provide context for emerging longer-term evidence on surface-modified flow diverters, including recently reported outcomes with the surface-modified FRED.^15,16^ Importantly, our study employed systematic 3D-DSA follow-up in most cases, which increases diagnostic precision but may increase detection of small residual filling, thereby modestly lowering complete occlusion estimates compared with less sensitive imaging modalities. Immediate postprocedural OKM grade was not predictive of long-term occlusion in this cohort.

Regarding safety at long-term follow-up (≥5 years), published data suggest an overall favorable profile, with few serious complications beyond the initial postprocedural period.^11–13,17–19^ In our cohort, symptomatic events were infrequent (ischemic 4.9%, hemorrhagic 1.0%), there were no treatment-related deaths during follow-up, and device-related findings were uncommon and almost exclusively asymptomatic. Collectively, these findings support the long-term safety and clinical acceptability of FRED for the management of intracranial aneurysms.

The dual-layer design of FRED is intended to combine flow disruption at the aneurysm neck with preservation of perforator and side-branch patency.^6^ Progressive neointimal coverage across the low-porosity inner layer may contribute to the increase in complete occlusion over time. Surface modifications in newer devices (eg, poly(2-methoxyethyl acrylate) in surface-modified FRED and phosphorylcholine in Pipeline Shield) may modulate early thromboinflammatory responses and device thrombogenicity^1,7,15,16^; however, their contribution to longer-term occlusion durability beyond the first year remains to be fully established.

### Limitations

Several limitations of this study should be acknowledged. First, although our cohort is large and prospectively collected, it was conducted at 2 centers with procedures performed by the same neurointerventional team, which may limit external generalizability. Second, despite the high rate of 5-year imaging follow-up and the systematic use of 3D-DSA in most cases, a minority of aneurysms did not undergo 5-year 3D-DSA and were assessed with CT or MR angiography. In addition, a small proportion did not complete 5-year imaging follow-up due to retreatment, death, or loss to follow-up. Third, as this was a prospective real-world registry, an independent core laboratory was not incorporated, which may introduce assessment bias; however, angiographic end points were assessed using predefined definitions with consensus review by at least 2 senior interventional neuroradiologists. Finally, although our methodology aimed to minimize selection bias, the findings should be interpreted within the context of real-world clinical practice.

### Conclusions

In summary, this prospective series provides the largest consecutive cohort to date with protocolized 5-year follow-up of patients treated with FRED for intracranial aneurysms. Our results confirm high and durable rates of aneurysm occlusion, with a low incidence of clinically significant complications. Notably, no cases of subarachnoid hemorrhage or treatment-related mortality occurred during 5 years of follow-up. These findings support the long-term safety and efficacy of FRED across different aneurysm types and locations and offer robust evidence to guide future research and clinical decision-making.

## ARTICLE INFORMATION

### Acknowledgments

The authors acknowledge their mentors, Jean-Jacques Merland, and Jacques Theron, for their pioneering contributions to the study and treatment of cerebrovascular disease.

### Disclosures

None.

### Supplemental Material

STROBE Checklist

## Supplementary Material

## References

[1] Gawlitza M Klisch J Kaiser DPO Linn J Pierot L Lobsien D. A systematic literature review and meta-analysis of the treatment of ruptured intracranial aneurysms with hydrophilic polymer and phosphorylcholine-coated flow diverters under single antiplatelet therapy. World Neurosurg. 2023;170:e791–e800. doi: 10.1016/j.wneu.2022.11.129 36462697 10.1016/j.wneu.2022.11.129

[2] Waqas M Dossani RH Alkhaldi M Neveu J Cappuzzo JM Lim J Khan A Lazarov V Monteiro A Davies JM. Flow redirection endoluminal device (FRED) for treatment of intracranial aneurysms: a systematic review. Interv Neuroradiol. 2022;28:347–357. doi: 10.1177/15910199211027991 34192977 10.1177/15910199211027991 PMC9185102

[3] Briganti F Leone G Marseglia M Mariniello G Caranci F Brunetti A Maiuri F. Endovascular treatment of cerebral aneurysms using flow-diverter devices: a systematic review. Neuroradiol J. 2015;28:365–375. doi: 10.1177/1971400915602803 26314872 10.1177/1971400915602803 PMC4757311

[4] Jesser J Alberalar ND Kizilkilic O Saatci I Baltacioglu F Özlük E Killer-Oberpfalzer M Vollherbst DF Islak C Cekirge SH. Safety and efficacy of the FRED Jr flow re-direction endoluminal device for intracranial aneurysms: retrospective multicenter experience with emphasis on midterm results. Front Neurol. 2021;12:722183. doi: 10.3389/fneur.2021.722183 34659086 10.3389/fneur.2021.722183 PMC8518710

[5] Hohenstatt S Ulfert C Herweh C Hilgenfeld T Schmitt N Schönenberger S Chen M Bendszus M Möhlenbruch MA Vollherbst DF. Long-term follow-up after aneurysm treatment with the flow redirection endoluminal device (FRED) flow diverter. Clin Neuroradiol. 2024;34:181–188. doi: 10.1007/s00062-023-01346-3 37833546 10.1007/s00062-023-01346-3 PMC10881684

[6] Guimaraens L Vivas E Saldaña J Llibre JC Gil A Balaguer E Rodríguez-Campello A Cuadrado-Godia E Ois A. Efficacy and safety of the dual-layer flow-diverting stent (FRED) for the treatment of intracranial aneurysms. J NeuroInterv Surg. 2019;12:521–525. doi: 10.1136/neurintsurg-2019-015371 31653756 10.1136/neurintsurg-2019-015371 PMC7231461

[7] Guimaraens L Saldaña J Vivas E Cifuentes S Balaguer E Mon D Macias-Gómez A Ois A Guisado-Alonso D Cuadrado-Godia E. Flow diverter stents for endovascular treatment of aneurysms: a comparative study of efficacy and safety between FREDX and FRED. J NeuroInterv Surg. 2025;17:e159–e159. doi: 10.1136/jnis-2023-021103 10.1136/jnis-2023-021103 38228386

[8] Shehata MA Ibrahim MK Ghozy S Bilgin C Jabal MS Kadirvel R Kallmes DF. Long-term outcomes of flow diversion for unruptured intracranial aneurysms: a systematic review and meta-analysis. J NeuroInterv Surg. 2023;15:898–902. doi: 10.1136/jnis-2022-019240 36150896 10.1136/jnis-2022-019240 PMC10033458

[9] Rios-Zermeno J Ghaith AK Perez-Vega C Greco E Michaelides L El Hajj VG Ortega-Ruiz OR Kumar JS Sandhu SJS Tawk RG. Pipeline embolization device for the treatment of unruptured intracranial saccular aneurysms: a systematic review and meta-analysis of long-term outcomes. Neurosurg Rev. 2024;47:813. doi: 10.1007/s10143-024-03040-5 39441223 10.1007/s10143-024-03040-5

[10] Abbas R Sweid A Salem MM Atallah E Naamani KE Amllay A Sioutas GS Sambangi A Yudkoff CJ Dougherty J. Predictors of occlusion, long-term outcomes, and safety in a cohort of 674 aneurysms treated with the pipeline embolization device. J Neurosurg. 2024;141:175–183. doi: 10.3171/2023.10.JNS231837 38181513 10.3171/2023.10.JNS231837

[11] Lylyk I Scrivano E Lundquist J Ferrario A Bleise C Perez N Lylyk PN Viso R Nella-Castro R Lylyk P. Pipeline embolization devices for the treatment of intracranial aneurysms, single-center registry: long-term angiographic and clinical outcomes from 1000 aneurysms. Neurosurgery. 2021;89:443–449. doi: 10.1093/neuros/nyab183 34098575 10.1093/neuros/nyab183 PMC8374967

[12] Becske T Brinjikji W Potts MB Kallmes DF Shapiro M Moran CJ Levy EI McDougall CG Szikora I Lanzino G. Long-term clinical and angiographic outcomes following pipeline embolization device treatment of complex internal carotid artery aneurysms: five-year results of the pipeline for uncoilable or failed aneurysms trial. Neurosurgery. 2017;80:40–48. doi: 10.1093/neuros/nyw014 28362885 10.1093/neuros/nyw014

[13] Naylor RM Abbasi M Brinjikji W Cloft HJ Kallmes DF Lanzino G. Long-term outcomes following pipeline embolization of unruptured aneurysms. Acta Neurochir. 2023;165:1891. doi: 10.1007/s00701-023-05619-1 37191722 10.1007/s00701-023-05619-1

[14] Dinc H Saatci I Oguz S Baltacioglu F Yildiz A Donmez H Belet U Onal B Andic C Koc O. Long-term clinical and angiographic follow-up results of the dual-layer flow diverter device (FRED) for the treatment of intracranial aneurysms in a multicenter study. Neuroradiology. 2021;63:943–952. doi: 10.1007/s00234-020-02627-1 33392735 10.1007/s00234-020-02627-1

[15] Roy JM Musmar B Karadimas S Lan M Koduri S Momin A McNulty A Paul A Zhang Y Puri AS. Multicenter comparative analysis of FRED-X, pipeline shield, and surpass evolve in treating intracranial aneurysms [published online October 25, 2025]. Interv Neuroradiol. 2025;15910199251384687. doi: 10.1177/15910199251384687 41137638 10.1177/15910199251384687 PMC12553543

[16] Roy JM El Naamani K Amaravadi C Majmundar S Mouchtouris N Paul AR Field NC Zhang Y Burkhardt JK Kühn AL. Long-term safety and efficacy of the FRED X flow diverter for intracranial aneurysms: a multicenter study of 154 patients. J Neurosurg. 2025;143:232–242. doi: 10.3171/2024.10.JNS241233 40020234 10.3171/2024.10.JNS241233

[17] Monteiro A Lim J Siddiqi M Donnelly BM Khawar W Baig A Turner RC Bouslama M Raygor KP Lai PMR. The first decade of flow diversion for intracranial aneurysms with the pipeline embolization device. Neurosurg Focus. 2023;54:E2. doi: 10.3171/2023.2.FOCUS22646 10.3171/2023.2.FOCUS22646 37127038

[18] Meyers PM Coon AL Kan P Dogan A Bain M Welch BG Ebersole K De Vries J Wakhloo AK Taussky P. Five-year results of the SCENT trial with surpass flow diverters to treat large or giant wide-neck aneurysms. Jo NeuroInterv Surg. 2025;17:1078–1082. doi: 10.1136/jnis-2024-022977 10.1136/jnis-2024-022977 40335285

[19] Lubicz B Van der Elst O Collignon L Mine B Alghamdi F. Silk flow-diverter stent for the treatment of intracranial aneurysms: a series of 58 patients with emphasis on long-term results. AJNR Am J Neuroradiol. 2015;36:542–546. doi: 10.3174/ajnr.A4143 25376806 10.3174/ajnr.A4143 PMC8013061

